# Optimizing tomato seedling growth with indigenous mangrove bacterial inoculants and reduced NPK fertilization

**DOI:** 10.3389/fpls.2024.1356545

**Published:** 2024-03-14

**Authors:** Soumaya Tounsi-Hammami, Munawwar Ali Khan, Aroosa Zeb, Aneesa Rasheed Anwar, Naman Arora, Muhammad Naseem, Sunil Mundra

**Affiliations:** ^1^ Department of Life and Environmental Sciences, College of Natural and Health Sciences at Zayed University, Dubai, United Arab Emirates; ^2^ Department of Life and Environmental Sciences, College of Natural and Health Sciences, Zayed University, Abu Dhabi, United Arab Emirates; ^3^ Department of Biology, College of Science, United Arab Emirates University, Abu-Dhabi, United Arab Emirates; ^4^ Khalifa Center for Genetic Engineering and Biotechnology (KCGEB), United Arab Emirates University, Al Ain, United Arab Emirates

**Keywords:** *Bacillus* sp., sustainable agriculture, biofertilization, nutrient management, *Avicennia marina*

## Abstract

The search for ecofriendly products to reduce crop dependence on synthetic chemical fertilizers presents a new challenge. The present study aims to isolate and select efficient native PGPB that can reduce reliance on synthetic NPK fertilizers. A total of 41 bacteria were isolated from the sediment and roots of mangrove trees (*Avicennia marina*) and assessed for their PGP traits under *in vitro* conditions. Of them, only two compatible strains of *Bacillus sp*ecies were selected to be used individually and in a mix to promote tomato seedling growth. The efficiency of three inoculants applied to the soil was assessed in a pot experiment at varying rates of synthetic NPK fertilization (0, 50, and 100% NPK). The experiment was set up in a completely randomized design with three replications. Results showed that the different inoculants significantly increased almost all the studied parameters. However, their effectiveness is strongly linked to the applied rate of synthetic fertilization. Applying bacterial inoculant with only 50% NPK significantly increased the plant height (44-51%), digital biomass (60-86%), leaf area (77-87%), greenness average (29-36%), normalized difference vegetation index (29%), shoot dry weight (82-92%) and root dry weight (160-205%) compared to control plants. Concerning the photosynthetic activity, this treatment showed a positive impact on the concentrations of chlorophyll a (25-31%), chlorophyll b (34-39%), and carotenoid (45-49%). Interestingly, these increases ensured the highest values significantly similar to or higher than those of control plants given 100% NPK. Furthermore, the highest accumulation of N, P, K, Cu, Fe, Zn, and Ca in tomato shoots was recorded in plants inoculated with the bacterial mix at 50% NPK. It was proven for the first time that the native PGP bacteria derived from mangrove plant species *A. marina* positively affects the quality of tomato seedlings while reducing 50% NPK.

## 1 Introduction

The world’s population is growing at an alarming rate and is expected to reach 9.1 billion by 2050. Consequently, the global food demand is expected to rise significantly, from 35% to 56% between 2010 and 2050 (Van Dijk et al., 2021). In the same period, the number of people at risk of hunger is projected to increase by 8% (Van Dijk et al., 2021), putting pressure on the agricultural sector to ensure food security. To achieve high crop production, synthetic fertilizers are commonly used in intensive agriculture and horticulture to correct soil nutrient deficiencies, particularly nitrogen, phosphorus, and potassium (Kong et al., 2018; Devi et al., 2022). Concerns over chemical residues in soil, water, and food have recently received significant attention due to their negative impact on environmental and human health (Akinnawo, 2023; Chataut et al., 2023). Furthermore, the consistent use of synthetic fertilizers presents a significant financial burden for farmers since fertilizer costs continue to rise (Majeed et al., 2015). Thus, agricultural and scientific communities are seeking innovative and sustainable management methods to improve crop yields and quality while safeguarding the environment and adapting to climate change.

Plant Growth Promoting bacteria (PGPB) are eco-friendly, low-cost natural resources that can be integrated to reduce the toxic effects of synthetic inputs and promote sustainable agriculture. PGP bacteria include different genera such as *Agrobacterium, Rhizobium, Azospirillum, Azotobacter, Bacillus*, and *Pseudomonas* (Boleta et al., 2020; Rosa et al., 2022; Tounsi-Hammami et al., 2022; Cirillo et al., 2023; Gen-Jiménez et al., 2023). These bacteria can act as biofertilizers through various mechanisms, including nitrogen fixation, nutrient solubilization, ammonium production, siderophore production and hormone production. Consequently, the use of PGPB can enhance plant nutrient uptake (Ijaz et al., 2021; Tsegaye et al., 2022) and increase the efficiency of synthetic fertilization (Mortinho et al., 2022; Asghar et al., 2023). The PGPB communities have great potential to reduce the dependency on synthetic fertilizers, such as nitrogen, phosphorus, and potassium, in both soil (Rosa et al., 2022; Tounsi-Hammami et al., 2022) and soilless conditions (Ikiz et al., 2024).

However, the success of PGPB inoculation depends on the efficiency of the selected strains, as well as its ability to adapt to environmental changes. Therefore, utilizing indigenous strains as biofertilizers has great potential, as they help maintain soil nutrient levels and can easily adapt to the local environment (Tounsi-Hammami et al., 2019). Given the lack of information on the impact of native PGP strains on reducing synthetic fertilizers in the United Arab Emirates, it was hypothesized that creating a consortium of indigenous PGPB could enhance plant nutrition and decrease farmers’ reliance on synthetic fertilizers. In this context, the goal of the present research is to isolate and characterize indigenous bacteria and assess the potential of elite PGP strains to substitute or reduce NPK fertilization while improving the growth and quality of tomato seedlings.

## 2 Materials and methods

### 2.1 Isolation, characterization and identification of native bacteria

#### 2.1.1 Sample collections

Samples of sediment and roots were collected in triplicates during March 2021 from Mangrove Beach in Umm Al Quwain, United Arabic Emirates (UAE) (25°32’06.0”N, 55°37’50.9”E), which is known as one of the UAE’s largest natural A. marina ecosystems (Samara et al., 2020). Soil sediments and roots were collected manually, using a sterilized spatula, within 10–15 cm of the wet area (25°32’ 07.8” N, 55°38 04.2” E) and dry area (25°32’ 15.9” N, 55°37 20.7” E) and were immediately placed individually in sterile zip-lock plastic bags. All samples were stored in ice-cold conditions (4°C) away from heat and sunlight and transported to the laboratory within 3 hours. All samples were stored at 4 °C until use. The physicochemical properties of the wet and dry sediment samples were recorded using standard procedures (**Table 1**).

**Table 1:** Dry and wet sediment characteristics at sampling locations.

	pH	EC (mS/cm)	OM (%)	N (mg/Kg)	P (mg/Kg)	K (mg/Kg)
Dry sediment	7.550	4.590	2.487	6792.493	288.867	735.367
Wet sediment	7.693	4.327	1.893	3593.267	458.833	808.890

Ec, Electric conductivity; OM, organic matter; N, total nitrogen; P, available phosphorous; K, potassium.

#### 2.1.2 Bacterial isolation

Briefly, 10 grams of dry/wet sediments and rhizosphere soil samples were collected from 15 cm depth at plant vegetation sites. One gram of soil sediment from dry and wet areas was aseptically transferred to a tube containing 9 mL of sterile distilled water and then shaken for 30 minutes. Afterwards, a series of decimal dilutions (10^−1^ to 10^−6^) of the soil suspensions were carried out in culture tubes. An aliquot of 100 µL of each dilution soil suspension was spread over a Jensen agar medium (HiMedia, Mumbai, India) and incubated at 28°C for 15 days for colony development. Single colonies were picked and repeatedly streaked on the same medium for purification. After isolation and purification, bacterial cultures were cultivated on LB media at 37°C for 48-72 hours.

For the isolation of root endophytes, about 1 gram of roots were surface sterilized with 70% ethanol for 1 minute, followed by soaking in a 25% sodium hypochlorite solution for three minutes to eliminate surface-contaminating microbes. Roots were rinsed in sterile distilled water to eliminate residual disinfectants and then air-dried in the laminar flow hood. The disinfected roots were excised and macerated in sterile distilled water using a mortar and pestle. About 1 ml of the sample was serially diluted in sterilized distilled water, and then 100 µL of each dilution aliquot was spread on Jensen agar medium. Plates were incubated at 28 °C for 15 days for colony development. Single colonies were picked and repeatedly streaked on the same medium for purification.

The pure bacteria isolated from wet and dry sediments and roots were maintained on nutrient broth agar slants, kept at 4°C, and −80 °C for long-term storage in nutrient broth adjusted with 20% (v/v) glycerol.

#### 2.1.3 Morphological and biochemical characterization

The isolates were examined for their morphological features (Aneja, 2003). Gram staining and biochemical tests were performed, including oxidase and catalase tests (Cappuccino and Sherman, 1992). API-20E micro-test system was used for eight strains. Bacterial isolates were tested for their ability to metabolize the 20 metabolites in the API-20E test kit according to the manufacturer’s instructions (BioMérieux, Marcy l’Etoile, France). The strips were incubated at 30°C, and the results were assessed after 24 and 48 h.

#### 2.1.4 Plant growth promotion traits

##### 2.1.4.1 Siderophore production

The secretion of siderophore was qualitatively analyzed using the Chrome Azurol S (CAS) method (Schwyn and Neilands, 1987). CAS agar plates were spot inoculated with each isolate, then incubated at 30°C for 7 days. After incubation, the appearance of an orange halo zone around bacterial isolates was considered positive (Louden et al., 2011). The experiment was performed in triplicate.

##### 2.1.4.2 Ammonia production (NH_3_)

Ammonia production was tested in peptone water following the Cappuccino and Sherman (1992) method. Ten ml of peptone water was inoculated with 100 µL (OD_600nm_ = 0.8) of a freshly cultured bacterial suspension, incubated at 30°C, and shaken at 150 rpm. An uninoculated medium served as the negative control. After 96 hours, 1 mL aliquots were taken and centrifuged at 10,000 rpm for 10 minutes. Then 0.5 ml of Nessler’s reagent was added to each supernatant. The development of a brown-to-yellow color indicated positive ammonia production.

##### 2.1.4.3 Indole acetic acid production

Quantitative estimation of IAA was performed on nutrient broth supplemented with tryptophan (0.2 mg ml^-1^) as a precursor of IAA, according to Gordon and Weber (1951). After 72 hours of incubation at 30°C with shaking at 150 rpm, bacterial cells were removed by centrifugation at 10000 g for 10 minutes at 4°C. The supernatants were filtered through 0.22 µm sterile syringe filters. One ml of each filtrate was mixed vigorously with 2 ml of Salkowski’s reagent consisting of 1 mL of 0.5 M FeCl_3_ and 50 mL of 35% HClO_4_ and allowed to stand at 30°C in the dark. After 20 minutes, the absorbance was recorded at 530 nm. The concentrations of IAA produced were estimated according to a standard curve using pure IAA (Sigma Aldrich, Overijse, Belgium) for concentrations in the 0–160 ppm range.

##### 2.1.4.4 Hydrogen cyanide production

The secretion of HCN was qualitatively analyzed by adapting the method of Lorck (1948). TSA medium was amended with 0.44% glycine, and 100 µL (OD_600nm_ = 0.6) of each strain was flooded on poured agar plates using a sterilized glass spreader. A sterilized Whatman filter paper was soaked in an alkaline picrate solution (0.25% picric acid in 1.25% sodium carbonate) for 1 minute and then placed at the top of the plate. Plates were sealed with parafilm and incubated at 30°C for 4 days. The paper reacts with HCN gas, and its colour changes from yellow to orange or brown, indicating HCN production.

##### 2.1.4.5 Potassium solubilization

The potassium solubilization was qualitatively evaluated on Alexandrov agar medium as described by Meena et al. (2015). Alexandrov agar plates were spot inoculated with each isolate and then incubated at 28°C for 7 days. The appearance of a clearing zone around the colonies indicated a positive potassium solubilization.

##### 2.1.4.6 Phosphate solubilization

The phosphate (P) solubilization was qualitatively estimated on Pikovskaya’s (PKV) agar medium (Pikovskaya, 1948). Plates were spot inoculated with each isolate and then incubated at 30°C for 7 days and observed for the development of the P solubilization zone around the colonies.

#### 2.1.5 Molecular identification by 16S rRNA gene amplification

Out of the 41 strains, only 8 underwent molecular identification. To isolate DNA for 16S rRNA gene amplification, Qiagen’s DNA extraction DNeasy^®^ bacterial isolation kit was used. The concentration of the extracted DNA was measured using a Nanodrop spectrometer (Implen-NP80). Then, the extracts were stored at -20°C for further analysis. The 16S rDNA gene amplification was conducted with the forward and reverse primers 27F and 1492R primers, respectively, with sequences 5′-AGAGTTGATCMTGGCTCGAG-3′ and 5′-GGTTACCTTGTTACGACTT-3′ (Frank et al., 2008). Sequencing was performed by Beijing Genomic Institute China (China). Subsequently, sequence similarities were determined using the BLAST program (https://www.blast.ncbi.nlm.nih.gov/Blast.cgi). Finally, all new sequences were deposited in the NCBI database with accession numbers OP600564 to OP600571. (https://www.ncbi.nlm.nih.gov/nuccore/?term=OP600564:OP600571%5baccn).

#### 2.1.6 Bacterial selection, compatibility test

Eight strains with multiple PGP traits were selected and tested for their compatibility with each other according to the methodology described by Prasad and Babu (2017). Two strains were grown in nutrient agar at 28°C for 3 days, then a first strain was streaked in the center of freshly prepared agar plates, and a second strain was streaked on either side of the central strain. Then, the Petri plates were incubated at 28°C for 72 hours, and an inhibition zone around the central strain was observed daily. For each pair of strains, three replications were performed.

### 2.2 Pot experiment

#### 2.2.1 Inoculum preparation

Only two strains were selected and used individually and in mix (M) to prepare the different inoculants. First, the selected strains were grown individually in glass tubes containing liquid nutrient broth medium on a rotating shaker (150 rpm) for 48 hours at 28°C to late exponential phase to obtain a final concentration of 10^9^ CFU mL^−1^. Then mix M was prepared by combining equal volumes of the individual bacterial suspensions before their application, corresponding to a final concentration of 10^9^ CFU mL^−1^.

#### 2.2.2 Experimental set up

To experiment, a commercial variety of tomatoes, called Shourouq, was used. The tomato seeds were surface sterilized by immersing in 70% ethanol for 1 minute, followed by soaking in a 25% sodium hypochlorite solution for 1 minute, then rinsed ten times in sterile distilled water and soaked for an additional 10 minutes in sterile distilled water to remove any remaining traces of the disinfectant. The effectiveness of this disinfection was ascertained by incubating ten seeds on nutrient broth agar plates at 28°C for 72 hours and checking for the absence of bacterial contamination.

The sterilized tomato seeds were immediately sown in 500 ml pots filled with a sterilized mix of vermiculite and peat (1:1, v/v). Two seeds were sown in each pot, and seven days after sowing (DAS), only one seedling per pot was kept. At this stage, inoculants were applied directly to the soil close to the stem at a rate of 5 mL per seedling (Trabelsi et al., 2011). Uninoculated plants were inoculated with the same amount of sterile nutrient broth and served as controls (C). Three levels of NPK synthetic fertilization were applied: 0, 50, and 100% of the recommended dose. The full dose of NPK synthetic fertilization was carried out using the commercial fertilizer Macro Greenmix 20:20:20 (N:P: K) at the rate of 3 grams/l as recommended by the manufacturer (Moncada et al., 2020). In all, 12 treatments were set up in three replications arranged in a completely randomized design (**Table 2**).

**Table 2:** Treatments used in the experiment, strain combinations, and levels of synthetic NPK fertilization.

T1: C-0%NPK	T5: C-50%NPK	T9: C-100%NPK
T2: S1-0%NPK	T6: S1-50%NPK	T10: S1-100%NPK
T3: S2-0%NPK	T7: S2-50%NPK	T11: S2-100%NPK
T4: M-0%NPK	T8: M-0%NPK	T12: M-100%NPK

C: uninoculated plants; S1: plants inoculated with strain S1=E1, S2: plants inoculated with Strain S2=E3, M, plants inoculated with the mix of the two strains: S1 and S2.

The experiment was carried out under a growth room chamber at 25/22^°^C day/night temperature, 16/8 hours light/dark period, and an average of 250 µmol m^−2^ s^−1^ light intensity as measured with a handheld spectrometer (LICOR, USA, model LI-180). The pots were rotated thrice weekly to ensure uniform growth conditions in the growth chamber.

#### 2.2.3 Plant and soil measurements

##### 2.2.3.1 Morphological and vegetative indices

The effects of different inoculants and synthetic fertilization on the morphological and vegetative of tomato plants were evaluated at 42 DAS using the Phenospex Scan device (PlantEye F500, Phenospex, Heerlen, The Netherlands). Various traits were measured, such as the digital biomass (mm^3^/plant) (DB), projected leaf area (mm^2^/plant), greenness average of the leaves, the normalized difference vegetation index (NDVI), and the plant senescence reflectance index (PSRI).

On the same day, the plant height (PH) was measured from the soil’s surface to the tip of the main stem for each treatment. The leaf numbers (LN) were determined for each plant. Then, tomato plants were harvested, and the stem diameter (SD) and root length (RL) were recorded. Then, plants were dried in an oven at 65°C until constant weight was achieved to determine their corresponding shoot and root dry weight (SDW, RDW).

##### 2.2.3.2 Physiological parameters

Leaf chlorophyll concentration was analyzed by estimating the content of chlorophyll a (Chla), chlorophyll b (Chlb), and carotenoid (Car) by spectrophotometry at 645 nm, 663 nm, and 470 according to the method of Torrecillas et al. (1984). The leaf’s relative water content (RWC) was determined as described by Turner (1986).

##### 2.2.3.3 Micro and macro-nutrient content

Dry shoots of tomato plants were powdered using a blade mill and then passed through a 40-mesh sieve. Plant tissue samples were used for mineral analysis. Total nitrogen (N) was determined by the method of Kjeldahl (1883). The phosphorus concentration was determined by colorimetric method at 840 nm (Pauwels et al., 1992). Furthermore, the potassium (K), sodium (Na), Magnesium (Mg), Calcium (Ca), Copper (Cu), Iron (Fe), and Zinc (Zn) concentrations were analyzed by inductively coupled plasma-optical emission spectrometry – ICP-OES (Model no. 700 series, Agilent Technology, Santa Clara, CA, USA) (Toselli et al., 2009).

##### 2.2.3.4 Soil microbial population

The soil microbial population was detected using the standard 10-fold dilution plating method. The data were expressed as the number of colony-forming units (CFU) per gram of soil (Somasegaran and Hoben, 1994).

### 2.3 Statistical analysis

The effect of bacterial inoculants, synthetic NPK fertilization, and their interactions on the various parameters measured in pot experiments was performed using the two-way analysis of variance (ANOVA) in accordance with the experimental design. The treatment means were compared using LSD tests at a significance level of 5%. The ranking of each treatment was denoted by letters. Pearson correlation coefficient analysis heat map was used to assess the relationships between all the measured parameters. All statistical analyses were performed using R statistical software version 3.5.

## 3 Results

### 3.1 Isolation, morphological and biochemical characterization

A total of 41 strains, all fast-growing, with 3 originating from interior roots, 3 from rhizosphere, and 11 and 24 originating respectively from dry and wet sediments. The morphological and biochemical characteristics of isolates deduced from the colony and microscopic features are presented in **Supplementary Table S1**. Most bacteria showed circular, cream, flat, and undulate colonies with variable sizes on nutrient broth agar plates. All bacteria tested positive for Gram test and catalase. The strains mostly tested positive for oxidase except SD2, SD3, SD6, SW1, SW4, SW12, and SW20. The data obtained using the API 20E biochemical identification system are shown in **Supplementary Table S2**. The results showed that all eight tested bacterial strains could consume ortho-nitrophenyl-b-galactoside (ONPG) and arginine. They were also able to perform fermentation of gelatine, glucose, and mannitol, except strain R2. However, these strains could not ferment sorbitol, rhamnose, sucrose, and melibiose. Only strain SW14 was able to consume citrate.

### 3.2 *In vitro* Plant Growth–Promoting traits

The results pertaining to various plant growth-promoting traits conducted are shown in **Table 3**. In total, 78% of strains were identified as ammonium producers. The highest production was observed in strain SD7. Moreover, eight strains, namely SW3, SW4, SW10, SW19, SW24, E1, E3, and R2, demonstrated a strong ability to produce ammonium.

**Table 3:** Plant growth–promoting abilities of the 42 native strains.

Strains	Ammonia production	IAA (mm)	P solubilization(mm)	K solubilization (mm)	Siderophore production (mm)	HCN
SD1	–	0.00	0.00	0.00	0.00	+
SD2	+	7.36ab ±0.03	7.00def ±0.00	0.00	0.00	+
SD3	+	3.10defgh ±0.04	7.00def ±0.00	0.00	4.667efgh ±1.15	+
SD4	–	0.00	0.00	0.00	0.00	+
SD5	++	2.51defgh ±0.12	8.83b ±0.29	0.00	1.67ghij ±0.58	+
SD6	+	0.00	0.00	0.00	2.00ghij ±1.00	+
SD7	++++	4.25cde ±0.42	7.00def ±0.00	25.00ab ±0.57	9.00bc ±2.00	+
SD8	+	1.96fgh ±0.51	7.33cdef ±0.59	22.50cd ±0.5	0.00	+
SD9	–	0.00	0.00	0.00	5.00defg ±2.00	+
SD10	+	2.34efgh ±0.13	7.67cd ±1.15	24.00abcd ±0.00	2.33fghij ±0.58	+
SD11	+	4.12cdefg ±0.18	7.50cde ±0.50	24.00abcd ±0.00	2.67fghij ±2.98	–
SW1	+	3.07defgh ±0.23	7.00def ±0.00	23.00bcd ±0.57	4.00fghij ±1.00	+
SW2	++	5.63bc ±0.03	7.00def ±0.00	22.00d ±0.57	9.00bc ±1.00	–
SW3	+++	3.61cdefgh ±0.13	9.00b ±0.00	0.00	12.67a ±3.06	+
SW4	+++	1.90cdefgh ±0.64	8.67b ±0.58	23.00bcd ±0.57	9.33ab ±0.58	+
SW5	–	0.00	0.00	0.00	1.33hij ±0.58	–
SW6	–	0.00	0.00	0.00	2.67fghij ±1.15	+
SW7	++	3.60cdefgh ±0.44	8.00c ±0.00	23.00bcd ±0.57	0.00	–
SW8	–	0.00	0.00	0.00	4.33efghi ±1.15	–
SW9	+	0.00	0.00	0.00	1.67ghij ±0.58	+
SW10	+++	0.00	0.00	0.00	3.67fghij ±2.08	+
SW11	–	0.00	0.00	0.00	3.67fghij ±1.15	–
SW12	++	3.34defgh ±0.26	7.00def ±0.00	0.00	8.33bcd ±1.15	–
SW13	++	2.83defgh ±0.41	7.33cdef ± 0.58	0.00	0.00	+
SW14	++	8.55a ±0.57	8.83d ±0.29	26.00a ±0.57	7.67bcde ±2.31	+
SW15	++	1.51h ±0.16	7.00def ±1.00	24.50abc ±0.00	0.00	+
SW16	+	4.14cdef ±0.00	6.67f ±1.15	0.00	0.00	+
SW17	++	0.00	0.00	0.00	1.67ghij ±0.58	+
SW18	+	0.00	0.00	0.00	0.00	+
SW19	+++	1.79h ±0.02	7.17def ±0.29	24.00abcd ±0.5	1.67ghij ±0.58	+
SW20	++	0.00	0.00	0.00	5.00defg ±1.00	+
SW21	+	0.00	0.00	0.00	1.00ij ±0.00	+
SW22	++	2.99defgh ±0.07	10.00a ±0.00	23.00bcd ±0.57	0.667j ±1.15	–
SW23	++	2.28efgh ±0.29	5.83g ±0.29	0.00	3.67fghij ±1.53	+
SW24	+++	0.00	0.00	0.00	9.00bc ±0.00	+
E1	+++	6.68cd ±0.11	7.00def ±0.00	25.00ab ±0.57	4.33efghi ±0.35	–
E2	++	0.00	0.00	0.00	3.33fghij ±1.53	+
E3	+++	1.87h ±0.12	7.17 def ± 0.29	23.00bcd ±0.57	5.67cdef ±0.58	+
R1	+	0.00	0.00	0.00	10.33ab ±-.58	+
R2	+++	2.38efgh ±0.34	8.67b ±0.58	22.50cd ±0.00	0.00	+
R3	++	2.00fgh ±0.07	6.83ef ±0.29	0.00	4.00fghij ±1.00	+

Values are the mean of three separate experiments in triplicates: ammonia production (based on the intensity of color): absent (-), weak (+), moderate (++), strong (+++), and highly strong (++++). Each value is the mean of three replicates. Different letters indicate significant differences according to the LSD test.

The quantitative estimation of the IAA production showed that only 24 strains (58%) could synthesize this phytohormone using tryptophane as a precursor. The amount of IAA production ranged from 1.51 to 8.55 ppm. The highest production was recorded by SW14, SD2, and SW2, producing 8.55 ppm, 7.36 ppm, and 5.63 of IAA, respectively. About 24 strains (58%) formed a visible halo, indicating a positive phosphate solubilizing activity, with a maximum recorded for SW22 (10 mm) and a minimum found for SW23 (5.83 mm). Only 15 strains were considered as potassium solubilizers, forming a visible halo around the colony. The highest values were found in SW14, with a diameter of 26 mm. In contrast, the smallest diameter was observed for strains SW2 (22 mm). Thirty-one strains were shown positive for siderophore production and formed an orange halo zone around the bacterial colony growing in CAS agar plate. The highest production was found in SW3, R1, and SW4, which showed the highest diameter, 12.63 mm, 10.33 mm, and 9.33 mm, respectively.

### 3.3 Molecular identification

A comparative sequence analysis of BLASTn search on NCBI revealed all the strains belonging to the phylum Firmicutes, class of Bacilli, and genus of *Bacillus* (**Table 4**). Three strains designated as SD7, SW7 and E3 were strongly related to *B. licheniformis* with a similarity compared to reference sequences ranging from 96.81% to 99.08. While three other strains designated as SD11, SW14, and SW22 could be affiliated respectively to *B. paralicheniformis*, *B. cabrialesii*, and *B. subtilis*. The remaining strains, designated as E1 and R2, shared 99.93 and 96.81% similarities with the most related strains, *B. wiedmannii* and *B. proteolyticus*, respectively.

**Table 4:** Taxonomic affiliation of selected eight strains based on nearly full-length 16S rRNA sequence analysis.

Strains	Sequence (bp)	Closest relative species	Phylum/class	Accession no.	Similarity (%)
SD7	1409	*Bacillus licheniformis*	Firmicuts/Bacilli	OP600567	99.08
SD11	1409	*Bacillus paralicheniformis*	Firmicuts/Bacilli	OP600568	100%
SW7	1409	*Bacillus licheniformis*	Firmicuts/Bacilli	OP600571	98%
SW14	1410	*Bacillus cabrialesii*	Firmicuts/Bacilli	OP600569	100%
SW22	1410	*Bacillus subtilis*	Firmicuts/Bacilli	OP600570	100%
E1	1395	*Bacillus wiedmannii*	Firmicuts/Bacilli	OP600564	99.93%
E3	1411	*Bacillus licheniformis*	Firmicuts/Bacilli	OP600565	96.81%
R2	1401	*Bacillus proteolyticus*	Firmicuts/Bacilli	OP600566	96.81%

### 3.4 Selection of compatible strains

According to the findings of the *in vitro* antagonistic assays (data not shown), it was determined that strains E3 and E1 are compatible and do not showcase any antagonistic effects toward each other. This was established by the absence of a growth inhibition zone at the intersection areas. As a result, it can be inferred that using these strains, either alone or in combination, is unlikely to cause any unwarranted interference.

### 3.5 Pot experiment

#### 3.5.1 Morphological parameters and vegetative indices

The analysis of variance indicates that bacterial inoculation, synthetic fertilization and their interactions had a significant effect on all the studied parameters except for GA, which was not influenced by interactions between the two factors (**Table 5** and **Supplementary Table S3**). It was found that the effectiveness of the bacterial inoculum varied depending on the level of NPK synthetic fertilization applied. Notably, tomato plant growth parameters showed significant improvement at 0% and 50% of synthetic fertilization compared to their respective controls C-0%NPK and C-50%NPK. Compared to control plants without NPK chemical fertilization, the inoculation with native strains S1, S2, and their mixture led to significant increases in DB (+159-162%), LA (+786-931%), GA (+47-66%) and NDVI (+41-50%). It is evident that these increases fell short of the expected maximum growth that can be attained with a complete dose of NPK fertilization. Indeed, the highest values were recorded for DB, LA, GA, and NDVI by applying the three inoculum and a half dose of NPK fertilization. Compared to the control plants at this treatment level, significant improvements amounting from 60 to 86% for DB, 77 to 87% for LA, 29 to 36% for GA and 29% for NDVI. Interestingly, at this treatment level, the growth parameters were statistically equivalent to, or higher than, those recorded in the control plants with 100% NPK fertilization. In contrast, at the full dose of NPK fertilization, inoculant application only had neutral effects on the growth parameters when compared to the application of synthetic NPK fertilizer alone.

**Table 5:** Effects of bacterial inoculation and synthetic fertilization on digital biomass (DB), leaf area (LA), greenness average (GA), the normalized difference vegetation index (NDVI), and plant senescence reflectance index (PSRI) of tomato plant at 42 DAS.

	DB (mm^3^/plant)	LA (mm^2^/plant)	GA	NDVI	PSRI
**C-0%NPK**	40.05c ±6.65	140.22f ±36	0.163d ±0.00	0.338e ±0.00	0.240a ±0.04
**S1-0%NPK**	104.81c ±11.82	2475.51e ±424	0.239c ±0.03	0.503d ±0.02	0.125bc ±0.04
**S2-0%NPK**	106.53c ±11.56	2153.84ef ±104	0.269bc ±0.04	0.508d ±0.04	0.133bc ±0.05
**M-0%NPK**	103.55c ±5.17	2129.21ef ±155	0.271dc ±0.07	0.478d ±0.06	0.155b ±0.01
**C-50%NPK**	849.93b ±94.96	8998.50d ±869	0.317b ±0.01	0.581c ±0.02	0.095cd ±0.01
**S1-50%NPK**	1517.69a ±187.67	15929.47ab ±1232	0.43a ±0.04	0.747ab ±0.01	0.042e ±0.02
**S2-50%NPK**	1361.64a ±295.06	16729.07a ±1195	0.41a ±0.03	0.749a ±0.01	0.043e ±0.02
**M-50%NPK**	1577.47a ±92.92	16792.07a ±2183	0.433a ±0.04	0.749a ±0.02	0.032e ±0.02
**C-100%NPK**	1439.63a ±194.13	13001.30c ±1482	0.393a ±0.01	0.699b ±0.03	0.051de ±0.02
**S1-100%NPK**	1426.45a ±155.94	13917.06bc ±1654	0.389a ±0.02	0.720ab ±0.02	0.036e ±0.02
**S2-100%NPK**	1584.69a ±318.59	12870.51c ±1357	0.418a ±0.05	0.730ab ±0.02	0.046e ±0.03
**M-100%NPK**	1541.07a ±256.68	13016.20c ±2477	0.399a ±0.04	0.727ab ±0.03	0.044e ±0.02
**Bacterial inoculation (BI)**	**	***	***	***	***
**Synthetic fertilization (SF)**	***	***	***	***	***
BI*SF	*	***	ns	***	*

C: uninoculated plants, S1: plants inoculated with strain E1, S2: plants inoculated with E3, M: plants inoculated with the mix of two strains, S1 and S2.

Each value is the mean of three replicates. Different letters indicate significant differences according to the LSD test.

Significance: ns = not significant; * significant at p < 0.05; ** significant at p < 0.01; *** significant at p < 0.001.

The plant senescence reflectance index (PSRI) was adversely affected by bacterial and synthetic fertilization and their interactions (**Table 5** and **Supplementary Table S3**). The highest PSRI readings were found in control plants not treated with synthetic fertilization, followed by inoculated plants at the same fertilizer level. Compared to control plants that received 50%NPK, bacterial inoculation caused a significant reduction in PSRI, with a reduction of 56% for S1, 55% for S2, and 66% for the bacterial mix M.

For their part, bacterial and synthetic fertilization positively affected the tomato plants’ LN, PH, RL, SD, SDW, and RDW, quantified at harvest (**Table 6** and **Supplementary Table S4**). The bacterial inoculants were more effective when the tomato plants received 50%NPK resulting in significant increases in the LN (+47-60%), PH (+44-51%), RL (+107-145%), SD (+79-84%), SDW (+82-92%), and RDW (+180-162%) compared to the control plants. Interestingly, at this treatment level, LN, RL, SD, SDW, and RDW were statistically higher than those recorded in the control plants with 100% N fertilization. It is important to highlight that for RL, the bacterial mix combined with 50% NPK reached an impressive length of 16.50 cm, which is significantly higher than that recorded for control plants receiving 100%NPK (10.99 cm).

**Table 6:** Effects of bacterial inoculation and synthetic fertilization on leaf number (LN), plant height (PH), root length (RL), stem diameter (SD), shoot dry weight (SDW), and root dry weight (RDW) of tomato plant at 42 DAS.

	LN	PH (cm/plant)	RL (cm/plant)	SD (mm/plant)	SDW (g/plant)	RDW (g/plant)
**C-0%NPK**	2.00g ±0.00	6.03d ±0.55	3.56f ±0.55	1.50d ±0.50	0.007d ±0.00	0.012d ±0.01
**S1-0%NPK**	3.33f ±0.58	12.13c ±0.32	7.60e ±0.36	2.50c ±0.50	0.083d ±0.00	0.134c ±0.04
**S2-0%NPK**	3.00f ± 0.00	12.17c ±0.28	7.13e ± 0.32	2.50c ±0.50	0.067d ±0.01	0.107c ±0.04
**M-0%NPK**	3.33f ± 0.57	12.00c ±0.50	7.80e ±0.264	2.83c ±0.29	0.077d ± 0.01	0.146c ±0.06
**C-50%NPK**	5.00e ± 0.00	15.83b ±0.76	6.73e ±0.35	3.17c ±0.29	0.499c ±0.09	0.156c ±0.05
**S1-50%NPK**	8.00ab± 0.00	23.13a ±0.32	15.07b ± 0.66	5.83a ±0.29	0.907ab ± 0.07	0.405a ±0.03
**S2-50%NPK**	7.33 bc ± 0.57	23.83a ±0.76	13.93c ± 1.00	5.67a ±0.76	0.924ab ±0.02	0.408a ±0.09
**M-50%NPK**	8.33a± 0.57	22.83a ±0.28	16.50a ± 0.87	5.83a ±0.29	0.958a ±0.03	0.475a ±0.09
**C-100%NPK**	6.33d± 1.15	23.83a ±0.28	10.99d ±0.68	4.67b ±0.58	0.905ab ±0.00	0.270b ±0.03
**S1-100%NPK**	6.67cd ± 0.57	23.47a ±0.55	11.33d ± 0.57	4.83b ±0.29	0.855b ±0.11	0.303b ±0.02
**S2-100%NPK**	6.33d ± 0.57	23.67a ±0.76	11.33d ± 0.57	4.83b ±0.76	0.836b ±0.07	0.277b ±0.03
**M-100%NPK**	6.67cd± 0.57	23.30a ±1.12	11.00d ± 1.00	4.50b ±0.50	0.865ab ±0.10	0.267b ±0.05
**Bacterial inoculation (BI)**	***	***	***	***	***	***
**Synthetic fertilization (SF)**	***	***	***	***	***	***
BI*SF	**	***	***	***	***	**

C: uninoculated plants, S1: plants inoculated with strain E1, S2: plants inoculated with E3, M: plants inoculated with the mix of two strains, S1 and S2.

Each value is the average of three replicates. Different letters indicate significant differences according to the LSD test.

Significance: ** significant at p < 0.01; *** significant at p < 0.001.

#### 3.5.2 Photosynthetic pigments and relative leaf water content

**Figure 1:**
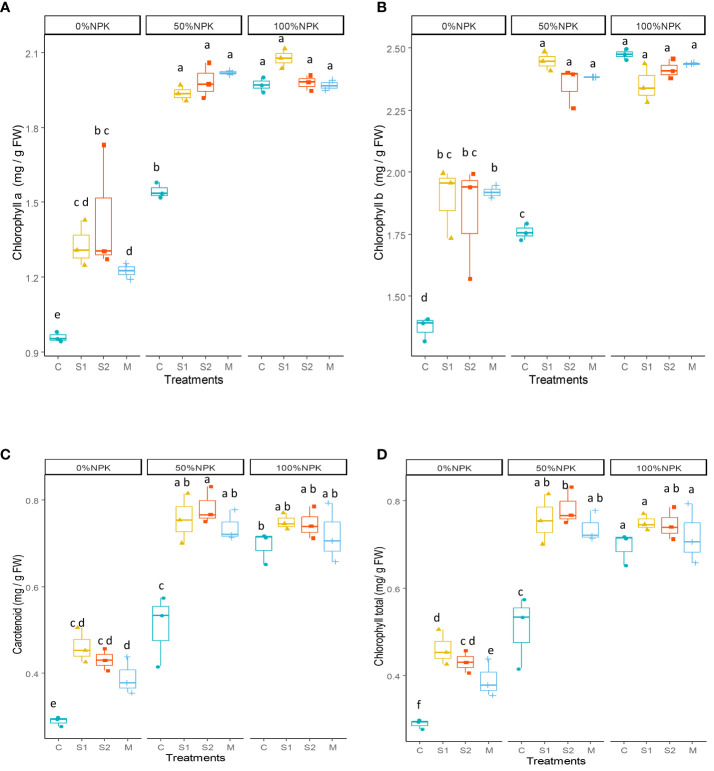
Effect of bacterial inoculation and synthetic fertilization on chlorophyll a **(A)**, chlorophyll b **(B)**, carotenoid **(C)**, total chlorophyll **(D)**. C: uninoculated plants, S1: plants inoculated with strain E1, S2: plants inoculated with E3, M: plants inoculated with the mix of two strains, S1 and S2. Each value is the mean of three replicates. Bars indicate the standard error of the mean. Different letters indicate significant differences according to the LSD test.

The photosynthetic pigments were affected by both bacterial inoculation and synthetic fertilization and the interactions between them (**Supplementary Table S5**). The results presented in **Figure 1** indicate that the application of different inoculants (S1, S2 and M) led to significant increases in chlorophyll a (25-28%), chlorophyll b (34-39%), carotenoid (45-49%), and total chlorophyll (31-33%) concentration when compared to control plants receiving only 50% NPK. Interestingly, these increases resulted in values that were either similar to or higher than those observed in the control plants receiving a full rate of NPK fertilization.

**Figure 2:**
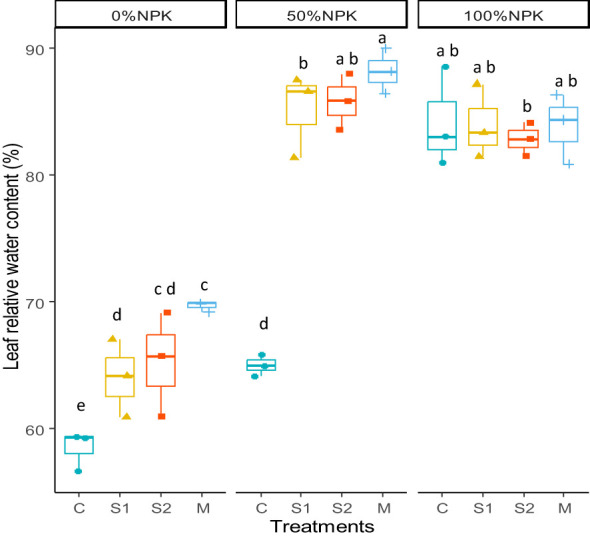
Effects of bacterial inoculation and synthetic fertilization on the leaf relative water content. C: uninoculated plants, S1: plants inoculated with strain E1, S2: plants inoculated with E3, M: plants inoculated with the mix of two strains, S1 and S2. Each value is the mean of three replicates. Bars indicate the standard error of the mean. Different letters indicate significant differences according to the LSD test.

The RWC was significantly influenced by the bacterial inoculants, the synthetic fertilization, and the interactions between the two factors (P < 0.001) (**Figure 2**, **Supplementary Table S5**). Compared to control plants, the RWC in leaves was significantly increased in response to bacterial inoculations at 0%NPK and 50%NPK. It was shown that the highest values were recorded in inoculated plants treated with 50%NPK.

#### 3.5.3 Micro and macro-nutrient content tomato shoots

The micro and macronutrient content in tomato shoots, including N, P, K, Na, Ca, Mg, Fe, Cu, and Zn, were presented in **Table 7**. The analysis of variance showed that the bacterial inoculation, the synthetic fertilization, and their interactions significantly affected the accumulation of all the studied nutrients except for Na content, which remained unaffected by the interactions between the two factors (**Supplementary Table S6**). In most cases, inoculated plants’ highest nutrient uptake was recorded, particularly with the bacterial mix treated with 50% NPK synthetic fertilization. At this level, the inoculation with native strains used individually and in mix significantly improved N (82-103%), P (90-101%), K (31-43%), Na (2-8%), Mg (4-13%), Ca (48-68%), Fe (62-70%), Cu (54-77%) and Zn (30-47%) compared with their respective control plants. Interestingly, these increases resulted in values statistically similar to or even higher than those observed in the control plants receiving a full dose of NPK synthetic fertilization.

**Table 7:** Effect of bacterial inoculation (BI) and synthetic fertilization (SF) on nitrogen (N), phosphorus (P), potassium (K), sodium (Na), magnesium (Mg), calcium (Ca), copper (Cu), iron (Fe) and zinc (Zn) contents of tomato shoots.

	N (mg/g DM)	P (mg/g DM)	K (mg/g DM)	Na (mg/g DM)	Mg (mg/g DM)	Ca (mg/g DM)	Cu (µg/g DM)	Fe (µg/g DM)	Zn (µg/g DM)
**C-0%NPK**	1.803c ±0.195	1.762f ±0.158	30.608c ±1.587	10.167b ± 0.057	33.892cde ±0.303	22.051e ±0.780	10.501h ± 0.635	33.667f ±3.210	98.904d ±2.080
**S1-0%NPK**	2.437b ±0.130	2.300de ±0.030	41.813b ±3.020	11.223ab ± 0.979	32.634e ±1.357	25.675d ±0.669	12.227gh± 0.484	45.000e ± 4.000	113.486cd ±1.987
**S2-0%NPK**	2.477b ±0.108	2.253e ±0.117	42.540b ±1.821	11.740ab ± 1.547	34.764bcde ±0.511	23.598de ±0.375	13.560fg ± 0.951	56.000d ±1.000	113.061cd ±0.908
**M-0%NPK**	2.717b ±0.057	2.607de ±0.040	43.753b ±1.995	12.427ab ± 1.01	35.603abcd ±1.471	23.400de ±1.320	15.673e ± 0.623	53.667d ±2.080	118.575c ±0.636
**C-50%NPK**	2.467b ±0.153	2.733d ±0.321	47.490b ±7.464	11.683ab ± 0.616	33.175de ±2.025	21.475e ±0.534	15.170ef ± 1.32	52.667d ±2.520	122.097c ±3.467
**S1-50%NPK**	4.867a ±0.493	5.267ab ±0.208	65.596a ±0.874	11.981a ± 1.673	34.754bcde ±0.145	31.988c ±3.624	23.363bc ± 1.152	85.333ab ±4.040	159.191b ±18.773
**S2-50%NPK**	4.500a ±0.529	5.200ab ±0.361	62.475a ±1.804	12.47ab ± 0.869	37.138ab ±3.082	34.521ab ±0.937	24.159b ± 0.545	89.000a ±3.360	183.079a ±11.167
**M-50%NPK**	5.000a ±0.500	5.500a ±0.265	68.213a ±0.266	12.648a ± 0.895	37.577a ±0.499	36.181a ±0.581	26.910a ± 1.449	90.000a ±3.000	179.072a ±9.577
**C-100%NPK**	4.633a ±0.379	4.933bc ±0.100	63.063a ±5.603	11.894a ± 0.492	35.470abcd ±0.779	33.714bc ±1.696	22.177cd ± 0.789	69.000c ±3.610	151.588b ±15.684
**S1-100%NPK**	4.533a ±0.351	5.000bc ±0.208	63.597a ±5.229	12.258a ± 1.751	36.198abc ±1.882	33.460bc ±1.989	21.344d ±0.560	87.667ab ±2.520	146.195b ±7.429
**S2-100%NPK**	4.533a ±0.451	4.733c ±0.436	67.481a ±2.163	11.900a ± 0.869	35.883abc ±2.211	32.660bc ±0.214	22.117cd ± 1.201	82.667b ±2.520	153.010b ±14.327
**M-100%NPK**	4.633a ±0.153	4.700c ±0.404	67.482a ±2.196	12.984a ±0.671	36.002abc ±0.194	33.401bc ±0.422	22.548bcd ± 1.867	87.667ab ±2.080	153.307b ±5.876
**Bacterial inoculation (BI)**	***	***	***	*	*	***	***	***	***
**Synthetic fertilization (SF)**	***	***	***	ns	*	***	***	***	***
BI*SF	***	***	***	ns	ns	***	***	***	***

C: uninoculated plants, S1: plants inoculated with strain E1, S2: plants inoculated with E3, M: plants inoculated with the mix of two strains, S1 and S2.

Each value is the mean of three replicates. Bars indicate the standard error of the mean. Different letters indicate significant differences according to the LSD test. Significance: ns = not significant; * significant at p < 0.05; *** significant at p < 0.001.

#### 3.5.4 Rhizosphere soil bacterial population at harvest

**Figure 3:**
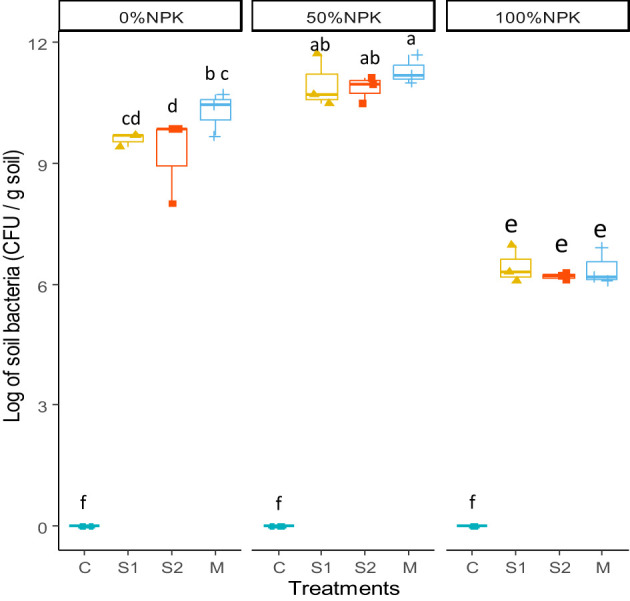
Effects of bacterial inoculation and synthetic fertilization on rhizosphere soil bacterial population at harvest. C: uninoculated plants, S1: plants inoculated with strain E1, S2: plants inoculated with E3, M: plants inoculated with the mix of two strains, S1 and S2. Each value is the mean of three replicates. Bars indicate the standard error of the mean. Different letters indicate significant differences according to the LSD test.

The colony-forming units of culturable soil bacteria were transformed into their logarithms and presented in **Figure 3**. The analysis of variance indicated significant effects of bacterial inoculation, synthetic fertilization, and their interactions on bacterial populations in the rhizosphere (**Supplementary Table S5**). The results clearly indicated that using a full dose of synthetic fertilizers negatively affects the microbial population of the rhizospheric soil (p < 0.001), resulting in significant decreases. However, when a half dose of NPK chemical fertilizers was used along with a bacterial inoculum, the rhizospheric microbial population reached the highest levels with statistical significance.

#### 3.5.5 Relationship between all the studied parameters

**Figure 4:**
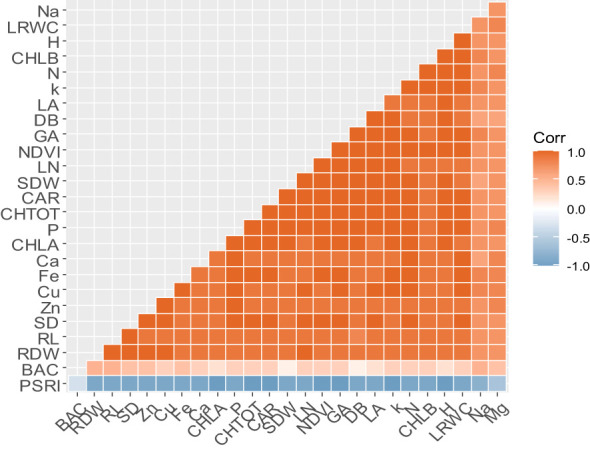
The Pearson correlation coefficient analysis heat map showing the correlation among all the studied parameters. DB, biomass; LA, leaf area; GA, greenness average; NDVI, the normalized difference vegetation index; PSRI, plant senescence reflectance index; LN, leaf number; PH, plant height; RL, root length; SD, stem diameter; SDW, shoot dry weight; RDW, root dry weight; CHLA, chlorophyll a; CHLB, chlorophyll b; CAR, carotenoid; CHLTOT, total chlorophyll; LRWC, leaf relative water content; BAC, bacterial population; N, nitrogen; P, phosphorus; K, potassium; Ca, calcium; Mg, magnesium; Fe, iron; Cu, copper; Zn, Zinc. Orange and blue gradients show positive and negative strength correlations; respectively.

The Pearson correlation analysis between all the studied parameters is presented in **Figure 4**. Strong and positive correlations were found among almost all the morphological parameters (DB, LA, LN, PH, RL, SD, SDW, and RDW), physiological parameters (GA, NDVI, CHLA, CLHB, CHLTOT, CAR, and LRWC), and nutrient accumulation (N, P, K, Na, Mg, Ca, Cu, Fe, and Zn). In contrast, all the studied parameters showed a strong negative correlation with PSRI. Regarding the bacterial population at harvest, the neutral correlation was shown with all the morphological, physiological parameters and nutrient accumulation.

## 4 Discussion

It is becoming increasingly popular to develop microbial consortia using bacteria with various plant growth-promoting (PGP) traits as an eco-friendly alternative to traditional farming techniques (Kalozoumis et al., 2021; Tounsi-Hammami et al., 2022). This study was designed to isolate native PGP bacteria to select suitable strains as potential biofertilizers for reducing the dependency on NPK synthetic fertilization in the United Arab Emirates (UAE). A total of 41 gram-positive and fast-growing bacteria were isolated from the wet-dry sediments and roots of *A. marina* trees (**Supplementary Table S1**). Most strains exhibited multiple PGP traits, including ammonia, IAA, siderophores production, P and K solubilization, and HCN production. Approximately 78% of native strains have been found to produce varying levels of ammonia, which acts as a nitrogen source for plant growth (Banik et al., 2019). Furthermore, excessive ammonia production can also prevent the colonization of pathogens on host plants and inhibit the germination of fungal spores (Singh and Parli, 2020). Considering the IAA production, the recorded amount ranged from 1.51 to 8.55 ppm with strains SW15 and SW14, respectively. The amounts of IAA detected in this study were similar to those reported by Barbaccia et al. (2022). The differences in the capacity of PGP bacteria to generate IAA, as presented in this study, may be due to a range of factors, such as the various biosynthetic pathways, the location of the involved genes, regulatory sequences, and the presence of enzymes that can convert active free IAA into conjugated forms (Patten and Glick, 1996). The IAA is widely recognized as the primary phytohormone that regulates many developmental processes. In low concentrations, IAA stimulates the elongation of primary roots. Conversely, elevated levels of IAA promote the growth of lateral and adventitious roots, facilitating water and nutrient absorption from the soil and indirectly promoting plant development (Naureen et al., 2017; Gowtham et al., 2022). Under *in vitro* conditions with iron limitation, 65% of the native strains were siderophore producers. It was reported that siderophores might help in the process of iron sequestration and solubilization, inhibiting the establishment of pathogenic (Sahu et al., 2021; Tripathi et al., 2022). Furthermore, our study discovered 24 and 15 native strains that effectively solubilize phosphate and potassium, respectively. These strains can convert insoluble P and K into plant-available forms, which is a crucial ability in areas where P and K are limiting factors for crop production (Bhat et al., 2022). Another mechanism of plant growth promotion, shown in our study, was the production of HCN, which is a volatile compound that plays a major role in the biocontrol process against phytopathogens (Sehrawat et al., 2022). Furthermore, Marques et al. (2010) reported a positive correlation between the generation of HCN and nitrogen accumulation, root and shoot elongation, and biomass production. These results highlighted the richness of the mangrove ecosystem dominated by *A. marina* sp. associated native bacteria with multiple PGP traits that can, directly and indirectly, promote plant growth, making them potential biofertilizers for agricultural crops in the UAE and MENA region.

After analyzing the 16S rRNA profile of the eight selected strains, it was found that they all belonged to the *Bacillus* group, specifically related to *B. licheniformis, B. Paralicheniformis, B. cabrialesii, B. subtilis*, and *B. wiedmannii*. Our findings are consistent with previous research showing that *Bacillus* is the most common group found in the *A. marina* ecosystem around the world, including China (Tong et al., 2019; Liu et al., 2022), Oman (Ali et al., 2017), and India (Pallavi et al., 2023). Most of these bacteria are known as beneficial bacteria with growth promotion abilities under optimum as well as under different abiotic and biotic stress conditions (Akhtar et al., 2020; Tsotetsi et al., 2021; Rojas-Solis et al., 2023). Among the most promising PGP bacteria, the *Bacillus* group is gaining more attention in agriculture thanks to its long shelf life and stability in harsh environments (Luo et al., 2022).

Two compatible strains, E1 and E3, with multi-PGP traits, were selected to be tested as biofertilizers for tomato plants at varying levels of NPK fertilization (0, 50, and 100% of the recommended dose). Results revealed that using these native PGP bacteria, either alone or mixed, significantly improved all the studied morphological parameters and vegetative indices in tomato plants, Shourouq variety, compared to control plants (**Tables 5**, **6**). However, strong interactions between bacterial inoculation and synthetic fertilization indicate that these PGP bacteria’ effectiveness depended on the synthetic NPK rate. Compared to the control plants, applying bacterial inoculant alone did not match the efficiency of synthetic NPK fertilizer in achieving maximum growth. This is consistent with several previous studies on other crops worldwide such as wheat (*Triticum turgidum* spp. *durum* L.) in Tunisia (Tounsi-Hammami et al., 2022), maize (*Zea mays* L.) in Brazil (Bueno et al., 2022), and cucumber (*Cucumis sativus* L.) in Italy (Scagliola et al., 2021).

It is important to note that combining native PGP bacteria with 50%NPK resulted in the highest values of DB, LA, LN, PH, RL, SD, SDW, and RDW. These values were statistically similar or even greater than those observed in control plants that received 100% NPK (**Tables 5**, **6**). This suggests that it’s possible to replace 50% of NPK synthetic fertilizers with selected native bacteria without negatively affecting the plant’s vegetative growth. The recorded improvement of morphological parameters is likely due to greater physiological activity compared to the control plants. Our findings show that applying native PGP bacteria along with 50%NPK resulted in significant increases in the relative leaf water content (**Figure 2**), indicating better water uptake and photosynthetic activity in plant tissues. Our findings supported the findings of a previous study by Yaghoubi Khanghahi et al. (2021) that bacterial inoculation can improve photosynthetic apparatus performance by positively impacting photosystem II functionality and chlorophyll pigment concentrations. This effect was reflected in the current study in higher GA, NDVI, and chlorophyll pigment concentrations, including the total chlorophyll, Chlorophyll a, Chlorophyll b, and carotenoids (**Table 5**; **Figure 1**). It may be suggested that inoculated plants could absorb more light energy to drive photosynthesis (Sahandi et al., 2019; Wu et al., 2019).

In contrast, when plants were given a full dose of synthetic NPK fertilization, the different bacterial inoculants showed no significant effect compared to control plants. According to Yan et al. (2019), synthetic fertilization did not affect the ability of beneficial bacteria to exhibit their PGP traits. However, the lack of any benefit from applying PGP bacteria in the present study may be explained by the significant decreases in the bacterial population recorded in the rhizospheric soil after applying 100%NPK (**Figure 3**). Our findings supported those of Yadav et al. (2009), who reported that the population of *G.diazotrophicus* in rhizospheric sugarcane was negatively affected by the addition of synthetic nitrogen. It was reported that high levels of synthetic inputs may enter the bacterial cells and disturb their metabolism, which decreases their abundance in rhizospheric soil (Reid et al., 2021), leading to significant reductions in root colonization (Bueno et al., 2022). It can be suggested that high levels of synthetic fertilizers lead to greater nutrient availability in rhizospheric soils, which may limit the plant’s need to interact with beneficial microorganisms in the rhizosphere (Meena et al., 2017). Interestingly, a half rate of synthetic NPK positively affected the bacterial population in tomato roots’ rhizosphere. It may be suggested that 50%NPK may support the growth of PGP bacteria and tomato plants equally (Zarei et al., 2012). Furthermore, the two selected native strains produced IAA, which induces significant increases in root development reflected in this study by increasing root length and biomass. It was reported previously that better root growth impacts soil microbiome via secreting more exudates, including sugars and organic acids. In response, soil microbes thrive and interact more frequently (Ye et al., 2020).

The boosting of plant growth observed in inoculated plants could be explained by the plants’ greater absorption of several nutrients from the soil (Anli et al., 2020). In fact, strong and positive correlations were found between nutrient accumulation, physiological parameters, and morphological parameters (**Figure 3**). Our results revealed that plants treated with the mix of native PGP strains and 50%NPK had considerably higher contents of N, P, K, Ca, Fe, and Zn (**Table 7**). Our results aligned with several previous research (Ye et al., 2020; Scagliola et al., 2021; Sritongon et al., 2023). A theoretical relationship between the *in vitro* and *in vivo* results could explain these findings. The IAA produced by the strains promotes the growth of root cortical cells, leading to an increase in the number of root tips and branches, allowing greater exploration of the soil and higher absorption of nutrients. Additionally, the ability of these PGP strains to produce ammonia and solubilize P and K under *in vitro* conditions could explain the observed enhancement of N, P and K accumulation in the tomato shoots. Furthermore, tomato shoots’ Fe, Zn, and Cu contents were significantly increased. This effect may be attributed to the ability of the two native strains to produce siderophores, known as iron-chelating compounds that enhance the uptake of various metals by plants (Scagliola et al., 2016; Prasad et al., 2019; Singh et al., 2022). Considering that crop productivity depends on early-stage seedling growth techniques, combining native PGP bacteria with 50% NPK should be adopted for farmers as well as for seedling nursery producers.

## 5 Conclusion

The present study demonstrates that using native PGP bacteria alone or in consortium as biofertilizers improves tomato seedling quality while reducing the global dependence on hazardous synthetic fertilizers. Although these results were obtained in partially controlled conditions, they suggest that bioinoculants could be used as a valid complement to synthetic fertilization for a more sustainable agricultural scenario. This simple approach can help farmers reduce cultivation costs while protecting the environment and human and soil health. However, further studies are required to evaluate the positive impact of these bacteria on the growth and productivity of other tomato varieties and different crops in large-scale field conditions and greenhouses.

## Data availability statement

The datasets presented in this study can be found in online repositories. The names of the repository/repositories and accession number(s) can be found below: NCBI database with accession numbers OP600564 to OP600571.

## Ethics statement

The manuscript presents research on animals that do not require ethical approval for their study.

## Author contributions

ST-H: Data curation, Formal analysis, Investigation, Supervision, Writing – original draft, Software. MK: Conceptualization, Funding acquisition, Methodology, Project administration, Resources, Supervision, Writing – review & editing, Validation. AZ: Formal analysis, Methodology, Writing – review & editing. AA: Investigation, Methodology, Validation, Writing – review & editing. NA: Formal analysis, Investigation, Methodology, Validation, Writing – review & editing. MN: Data curation, Formal analysis, Methodology, Resources, Validation, Writing – review & editing. SM: Funding acquisition, Resources, Supervision, Visualization, Writing – review & editing.
