# Supplementary material for: Optimizing tomato seedling growth with indigenous mangrove bacterial inoculants and reduced NPK fertilization
Source: Front Plant Sci. 2024 Mar 14;15:1356545. doi: 10.3389/fpls.2024.1356545 (PMC10973150; doi:10.3389/fpls.2024.1356545)
Supplement: Supplementary file 1 [file DataSheet_1.docx]

**Supplementary Material**

| **Table S1:** Morphological and biochemical characterization of the strains isolated from rhizosphere and roots of mangrove trees in UAE | | | | | | | | | |
| --- | --- | --- | --- | --- | --- | --- | --- | --- | --- |
| **Strain** | **Origin** | **Shape** | **Colour** | **Elevation** | **Margin** | **Diameter (mm)** | **Gram test** | **Oxidase** | **Catalase** |
| SD1 | DS | Circular | Cream | Flat | Undulate | 2 | + | + | + |
| SD2 | DS | Circular | Cream | Flat | Undulate | 3 | + | - | + |
| SD3 | DS | Circular | Cream | Raised | Entire | 4 | + | - | + |
| SD4 | DS | Circular | Cream | Flat | Undulate | 5 | + | + | + |
| SD5 | DS | Irregular | Cream | Flat | Undulate | 3 | + | + | + |
| SD6 | DS | Irregular | Cream | Flat | Undulate | 3 | + | - | + |
| SD7 | DS | Irregular | Cream | Flat | Undulate | 5 | + | + | + |
| SD8 | DS | Circular | Cream | Flat | Undulate | 4 | + | + | + |
| SD9 | DS | Circular | Cream | Flat | Undulate | 4 | + | + | + |
| SD10 | DS | Circular | Cream | Flat | Entire | 4 | + | + | + |
| SD11 | DS | Circular | Cream | Flat | Undulate | 4 | + | + | + |
| SW1 | WS | Circular | Cream | Flat | Undulate | 3 | + | - | + |
| SW2 | WS | Circular | Opaque | Flat | Entire | 3 | + | + | + |
| SW3 | WS | Circular | Cream | Flat | Undulate | 2 | + | + | + |
| SW4 | WS | Circular | Cream | Flat | Undulate | 3 | + | - | + |
| SW5 | WS | Circular | Cream | Raised | Undulate | 3 | + | + | + |
| SW6 | WS | Circular | Cream | Raised | Undulate | 3 | + | + | + |
| SW7 | WS | Circular | Cream | Raised | Undulate | 2 | + | + | + |
| SW8 | WS | Irregular | Opaque | Flat | Undulate | 6 | + | + | + |
| SW9 | WS | Circular | Opaque | Flat | Undulate | 7 | + | + | + |
| SW10 | WS | Circular | Cream | Flat | Undulate | 5 | + | + | + |
| SW11 | WS | Circular | Cream | Raised | Undulate | 3 | + | + | + |
| SW12 | WS | Circular | Cream | Flat | Entire | 5 | + | - | + |
| SW13 | WS | Circular | Cream | Flat | Undulate | 3 | + | + | + |
| SW14 | WS | Circular | Cream | Flat | Entire | 4 | + | + | + |
| SW15 | WS | Circular | Cream | Flat | Undulate | 4 | + | + | + |
| SW16 | WS | Circular | Cream | Flat | Undulate | 2 | + | + | + |
| SW17 | WS | Circular | Cream | Flat | Undulate | 5 | + | + | + |
| SW18 | WS | Circular | Cream | Flat | Undulate | 5 | + | + | + |
| SW19 | WS | Irregular | Cream | Raised | Undulate | 3 | + | + | + |
| SW20 | WS | Irregular | Cream | Flat | Undulate | 4 | + | - | + |
| SW21 | WS | Circular | Opaque | Flat | Undulate | 4 | + | + | + |
| SW22 | WS | Circular | Cream | Flat | Entire | 5 | + | + | + |
| SW23 | WS | Irregular | Cream | Flat | Undulate | 7 | + | + | + |
| SW24 | WS | Circular | Cream | Flat | Undulate | 7 | + | + | + |
| E1 | MR | Irregular | Cream | Flat | Undulate | 4 | + | + | + |
| E2 | MR | Irregular | Cream | Flat | Undulate | 1 | + | + | + |
| E3 | MR | Irregular | Cream | Flat | Undulate | 4 | + | + | + |
| R1 | MRH | Circular | Cream | Flat | Entire | 2 | + | + | + |
| R2 | MRH | Circular | Cream | Flat | Entire | 3 | + | + | + |
| R3 | MRH | Circular | Cream | Raised | Entire | 5 | + | + | + |

DS: dry sediment, WS: wet sediment, MR: mangrove roots, MRH: mangrove rhizosphere

Table S2: Biochemical Characterization of selected eight PGPB strains using API-Kit

|  | **SD7** | **SD11** | **SW7** | **SW14** | **SW22** | **E1** | **E3** | **R2** |
| --- | --- | --- | --- | --- | --- | --- | --- | --- |
| ONPG* (Beta-galactosidase) | + | + | + | + | + | + | + | - |
| Arginine (Arginine dihydrolase) | + | + | + | + | + | + | + | + |
| Lysine (Lysine decarboxylase) | - | - | - | - | - | - | - | - |
| ODC | - | - | - | - | - | - | - | - |
| Citrate (Citrate utilization) | - | - | - | + | - | - | - | - |
| Na thiosulfate (H2S production) | - | - | - | - | - | - | - | - |
| Urea (Urea hydrolysis) | - | - | - | - | - | - | - | - |
| Tryptophan (Deaminase) | + | + | + | + | + | + | + | + |
| Indole (Indole Production) | + | - | + | - | + | + | + | + |
| Na pyruvate (Acetoin production) | - | + | + | + | - | - | - | - |
| Charcoal gelatin (Gelatinase) | + | + | + | + | + | + | + | - |
| Glucose (Fermentation/oxidation) | + | + | + | + | + | + | + | - |
| Mannitol (Fermentation/oxidation) | + | + | + | + | + | + | + | - |
| Inositol (Fermentation/oxidation) | + | - | + | - | + | + | - | - |
| Sorbitol (Fermentation/oxidation) | - | - | - | - | - | - | - | - |
| Rhamnose (Fermentation/oxidation) | - | - | - | - | - | - | - | - |
| Sucrose (Fermentation/oxidation) | - | - | - | - | - | - | - | - |
| Melibiose (Fermentation/oxidation) | - | - | - | - | - | - | - | - |
| Amygdalin (Fermentation/oxidation) | + | + | - | + | + | + | + | - |
| Arabinose (Fermentation/oxidation) | - | + | + | - | + | + | + | - |

**+: positive activity, -: negative activity**

**Table S3:** Two-way analyses of variance (means of squares and their associated probabilities) of the effects of bacterial inoculation (BI), synthetic fertilization (SF) and their interactions (BI*SF) on digital biomass (DB), leaf area (LA), greenness average (GA), normalized difference vegetation index (NDVI), plant senescence reflectance index (PSRI)

|  | **Df** | **DB** | **LA** | **GA** | **NDVI** | **PSRI** |
| --- | --- | --- | --- | --- | --- | --- |
| **BI** | 3 | 494016** | 73150000*** | 0.036*** | 0.094*** | 0.021*** |
| **SF** | 2 | 14191034*** | 1199000000*** | 0.213*** | 0.526*** | 0.106*** |
| **BI*SF** | 6 | 552820* | 66650000*** | 0.015^ns^ | 0.029*** | 0.011*** |
| **Residuals** | 24 | 739337 | 43210000 | 0.033 | 0.019 | 0.017 |

Significance: ns = not significant; * significant at p < 0.05; ** significant at p < 0.01; *** significant at p < 0.001.

**Table S4:** Two-way analyses of variance (means of squares and their associated probabilities) of the effects of bacterial inoculation (BI), synthetic fertilization (SF) as well as their interactions (BI*SF) on leaf number (LN), plant height (PH), root length (RL), stem diameter (SD), shoot dry weight (SDW) and root dry weight (RDW).

|  | **Df** | **LN** | **PH** | **RL** | **SD** | **SDW** | **RDW** |
| --- | --- | --- | --- | --- | --- | --- | --- |
| **BI** | 3 | 14.310*** | 130.600*** | 123.420*** | 10.720*** | 0.152*** | 0.130*** |
| **SF** | 2 | 125.390*** | 1161.600*** | 271.200*** | 54.430*** | 4.940*** | 0.428*** |
| **BI*SF** | 6 | 9.940** | 78.600*** | 82.580*** | 7.900*** | 0.288*** | 0.084** |
| **Residuals** | 24 | 7.330 | 8.700 | 10.170 | 5.830 | 0.084 | 0.062 |

Significance: ns = not significant; * significant at p < 0.05; ** significant at p < 0.01; *** significant at p < 0.001.

**Table S5:** Two-way analyses of variance (means of squares and their associated probabilities) of the effects of bacterial inoculation (BI), synthetic fertilization (SF) and their interactions (BI*SF) on chlorophyll a (Chla), chlorophyll b (Chlb), carotenoid (Car), chlorophyll tot (Chl tot), leaf water relative content (WRC) and soil microbial populations (SMP).

|  | **Df** | **Chla** | **Chlb** | **Car** | **Chl tot** | **LWRC** | **SMP** |
| --- | --- | --- | --- | --- | --- | --- | --- |
| **BI** | 3 | 0.547*** | 0.867*** | 0.149*** | 2.735*** | 771.600*** | 551.100*** |
| **SF** | 2 | 3.995*** | 2.825*** | 0.817*** | 13.494*** | 1670.500*** | 78.700*** |
| **BI*SF** | 6 | 0.285*** | 0.671*** | 0.052** | 1.605*** | 481.700*** | 26.900*** |
| **Residuals** | 24 | 0.171 | 0.188 | 0.050 | 0.040 | 137.900 | 5.100 |

Significance: ns = not significant; * significant at p < 0.05; ** significant at p < 0.01; *** significant at p < 0.001.

**Table S6** Two-way analyses of variance (means of squares and their associated probabilities) of the effects of bacterial inoculation (BI), synthetic fertilization (SF) and their interactions (BI*SF) on nitrogen (N), phosphorus (P), potassium (K), sodium (Na), magnesium (Mg), copper (Cu), iron (Fe) and zinc (Zn) contents of tomato shoots

|  | **Df** | **N** | **P** | **K** | **Na** | **Mg** | **Ca** | **Cu** | **Fe** | **Zn** |
| --- | --- | --- | --- | --- | --- | --- | --- | --- | --- | --- |
| **BI** | 3 | 7.090*** | 7.360*** | 625.000*** | 9.534* | 30.910* | 158.100*** | 157.100*** | 3805.000*** | 4024.000*** |
| **SF** | 2 | 34.050*** | 51.29*** | 4536.000*** | 5.645ns | 19.560* | 608.000*** | 682.800*** | 8971.000*** | 16734.000*** |
| **BI*SF** | 6 | 6.850*** | 9.160*** | 536.000*** | 2.765ns | 22.990ns | 256.800*** | 118.000*** | 680.000*** | 3709.000*** |
| **Residuals** | 24 | 2.72 | 1.590 | 294.000 | 27.629 | 54.600 | 49.300 | 26.500 | 225.000 | 2016.000 |

Significance: ns = not significant; * significant at p < 0.05; ** significant at p < 0.01; *** significant at p < 0.001.
